# A case of IgG4-related hepatic inflammatory pseudotumor replaced by an abscess after steroid treatment

**DOI:** 10.1186/s12876-016-0504-6

**Published:** 2016-08-02

**Authors:** Masayuki Shibata, Hiroyuki Matsubayashi, Tsuyoshi Aramaki, Katsuhiko Uesaka, Naoyuki Tsutsumi, Keiko Sasaki, Hiroyuki Ono

**Affiliations:** 1 Division of Endoscopy, Shizuoka Cancer Center, 1007 Shimonagakubo, Nagaizumi, Suntogun, Shizuoka 411-8777 Japan; 2 Division of Interventional Radiology, Shizuoka Cancer Center, Nagaizumi, Suntogun, Shizuoka 411-8777 Japan; 3 Division of Hepato-Biliary-Pancreatic Surgery, Shizuoka Cancer Center, Nagaizumi, Suntogun, Shizuoka 411-8777 Japan; 4 Division of Infectious Diseases, Shizuoka Cancer Center, Nagaizumi, Suntogun, Shizuoka 411-8777 Japan; 5 Division of Pathology, Shizuoka Cancer Center, Nagaizumi, Suntogun, Shizuoka 411-8777 Japan

**Keywords:** Hepatic inflammatory pseudotumor, IgG4-related disease, Steroid, Abscess, Liver biopsy

## Abstract

**Background:**

Hepatic inflammatory pseudotumor (IPT) is a rare disease which often mimics a malignant tumor and is therefore often misdiagnosed and surgically resected. Recently, a concept of IgG4-related diseases (IgG4-RD) has been proposed that is becoming widely recognized and includes IgG4-related hepatic IPT. Corticosteroids are widely accepted as the standard treatment.

**Case presentation:**

A 72-year-old Japanese man, who had been followed for ten years after surgery and chemotherapy for treatment of hilar and lower bile duct cancers, developed intermittent fever and abdominal pain and visited this hospital. Blood examinations revealed an inflammatory reaction, worsened glucose intolerance, and an increased level of serum IgG4 (137 mg/dL). Computed tomography (CT) revealed a 5 cm-sized mass in hepatic segment 7. Because of his cancer history, not only was a benign mass suspected, but there was also the possibility of a recurrent biliary malignancy. Liver biopsy was performed and the histology met the criteria for IgG4-related IPT. Corticosteroid therapy was initiated and his symptoms quickly resolved. However, two months later, a repeat CT demonstrated that the hepatic mass had been replaced by an abscess. The abscess was initially refractory, despite tapering corticosteroid treatment, controlling diabetes by intensive insulin therapy, administration of antibiotics, and percutaneous abscess drainage. Finally, after six months, the condition resolved.

**Conclusion:**

The diagnosis of hepatic IPT is sometimes difficult. To differentiate it from a malignant tumor, histological examination is necessary. Although corticosteroids are recognized as the standard therapy, unexpected and critical complications can develop in cases of IgG4-related hepatic IPT.

## Background

Hepatic inflammatory pseudotumor (IPT) is a rare entity that has been thought to be a composition which develops through a variety of pathogeneses, such as infections with a virus [1] or bacteria [2, 3], congenital diseases [4], gallstones [5], and chronic biliary inflammation [6]. This disease mimics a malignant tumor on clinical images, and is therefore often misdiagnosed and surgically resected. The concept of this disease has been further presented after the disclosure of the concept of immunoglobulin G4 (IgG4)-related hepatic IPT [7]. Recently, a consensus statement of IgG4-related diseases (IgG4-RD) has been published, including IgG4-related hepatic IPT, and corticosteroids are the most suitable treatment for this disease [8–10]. The current report presents a rare case of IgG4-related hepatic IPT that developed into an abscess after corticosteroid therapy and needed long-term therapy.

## Case presentation

A Japanese man was being followed after he underwent left hepatectomy and pancreatoduodenectomy for a mucin-producing hilar cholangiocarcinoma (the postoperative pathological diagnosis was well differentiated papillary adenocarcinoma, T2, N0, M0, stage II by the International Union Against Cancer TNM classification) and lower bile duct carcinoma (the postoperative pathological diagnosis was well differentiated papillary adenocarcinoma, T1, N0, M0, stage I) 10 years ago when he was 62 years of age. He had also undergone chemoradiation therapy during the year following surgery (external radiation: total 50.4 Gy for four weeks, chemotherapy: 310 mg/day of continuous infusion of 5-fluorouracil for three years). Follow up was continued after this chemoradiation management, and the patient’s course was uneventful without recurrence, except for new onset of diabetes after surgery [serum hemoglobin A_1c_ (HbA_1c_): 6.4–7.4 %, normal range: 4.6–6.2 %].

**Fig. 1:**
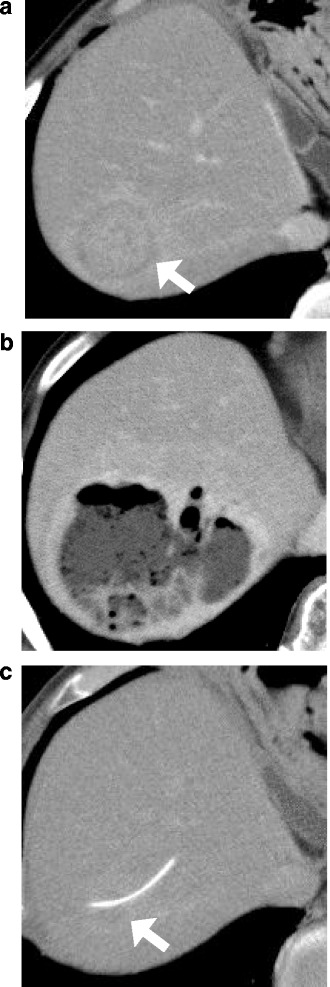
Enhanced computed tomography (CT) at initial diagnosis (**a**), two months after steroid initiation (**b**), and after percutaneous drainage placement (**c**). Initial CT showing a 5 cm-sized hepatic mass in segment 7 (**a**). Following CT after steroid initiation showing a 9.5 cm, multilocular abscess replacing the inflammatory pseudotumor and containing fluid and gas with marginal enhancement (**b**). CT after percutaneous drainage placement showing a minimized abscess lesion (**c**)

**Fig. 2:**
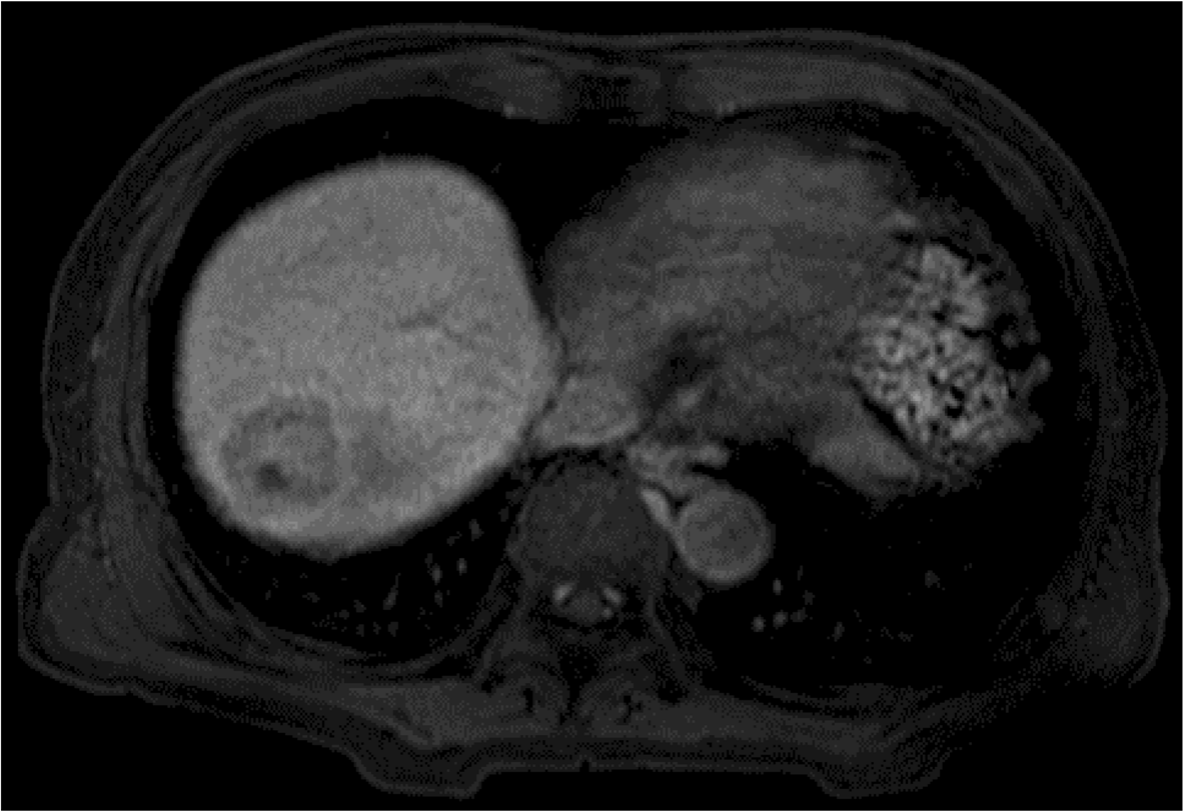
Magnetic resonance imaging (MRI) at 15 min since contrast injection. MRI enhanced with gadoxetate sodium showing a faint uptake of the contrast medium at the central area of the hepatic mass

**Fig. 3:**
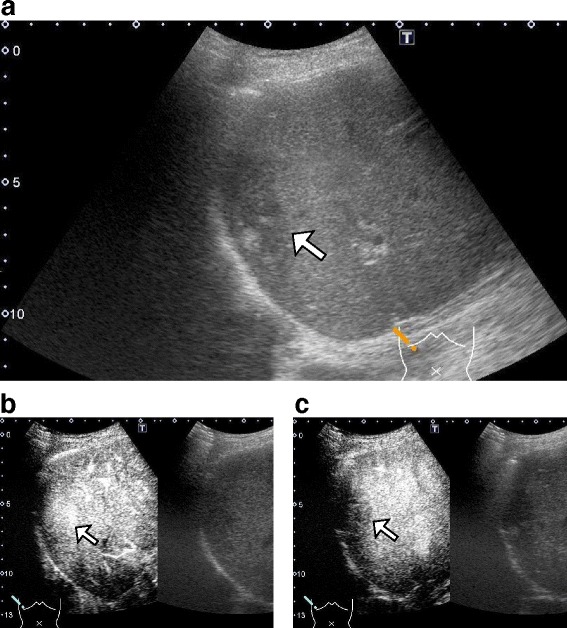
Enhanced ultrasonography using perfluorobutane microbubble. An irregular-margin of low echoic mass depicted by B-mode image (**a**). The lesion infused by microbubble at 10 s after contrast injection (**b**). Defected microbubble enhancement at 20 s (**c**)

**Fig. 4:**
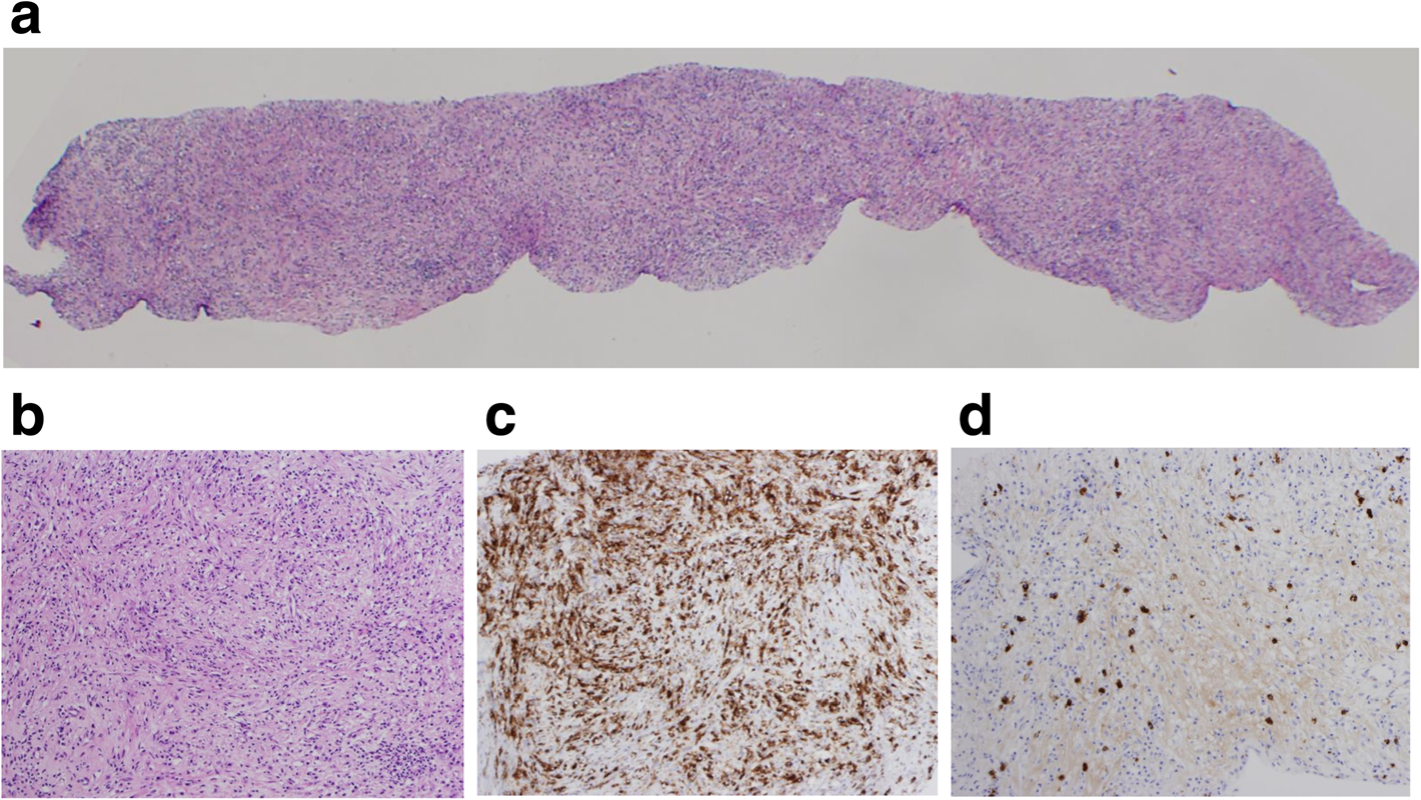
Histological view of the hepatic biopsy. Low power view (**a**) and high power view (**b**) of hematoxylin and eosin (HE) staining. CD68 protein diffusely expressed in numerous number of macrophages and storiform fibrosis (CD68) (**c**). Increased number of IgG4-positive cells infiltrating in the fibrous tissue (IgG4) (**d**)

At the age of 72, this patient presented to the hospital with complaints of intermittent fever and abdominal pain. His laboratory data showed increasing levels of white blood cells (WBCs) (17500 /μL, normal: 3900–9800 /μL), alkaline phosphatase (421 U/L, normal: 115–359 U/L), C-reactive protein (CRP) (8.99 mg/dL, normal: <0.3 mg/dL), HbA_1c_ (8.8 %), and IgG4 (137 mg/dL, normal: 48–105 mg/dL). Serum levels of hepatobiliary enzymes, carcinoembryonic antigen, carbohydrate antigen 19-9, and α-fetoprotein were within the normal range. Computed tomography (CT) revealed a 5-cm mass lesion in hepatic segment 7 (Fig. 1a), but no remarkable findings in the most commonly affected organs of IgG4-RD, which include the pancreas, abdominal aorta, and kidney. Based on magnetic resonance imaging enhanced with gadoxetate sodium (Fig. 2) and ultrasonography enhanced with perfluorobutane microbubble (Fig. 3), we considered the possibility of a hepatic abscess and a metastatic recurrence of cholangiocarcinoma. Ultrasonography (US)-guided percutaneous needle biopsy (SuperCore™, 18 gauge, 15 cm, Sheeman, Osaka, Japan) was performed on this hepatic mass. Histopathology of the biopsy specimen demonstrated abundant inflammatory cell infiltration surrounded by dense fibrous tissue, or so-called storiform fibrosis (Fig. 4a, b, c). No cancer cells were recognized. On immunohistochemistry, >20 of IgG4-positive plasma cells were counted per high power field (HPF) (Fig. 4d) and the ratio of IgG4/IgG was 17.3 %. These clinicopathological findings met the criteria of a possible IgG4-RD [9, 10] and the hepatic mass was diagnosed as IgG4-related hepatic IPT.

To avoid aggravation of the patient’s diabetes mellitus, corticosteroids were initially not offered and he was treated with antibiotics, non-steroidal anti-inflammatory drugs, and ursodeoxycholic acid. However, the patient’s symptoms continued, mandating that steroid therapy be considered. The patient was offered the opportunity to provide informed consent about expected outcomes and possible adverse events. Oral steroid therapy was started on admission to control blood glucose level by intensive insulin therapy. The dose of prednisolone was 30 mg/day for the first week, tapered by 5 mg per week until the dose reached 10 mg/day, and further tapered in the decelerated pitch (1 mg per week). Concerning about exacerbation of his hyperglycemia, we accelerated the steroid tapering. After treatment with oral steroids, he had no symptoms and his laboratory data reached the nearly normal levels (serum hepatic enzymes was normalized and CRP level declined to 1.02 mg/dL) and the shrinkage of the IPT was recognized by US. However, after three months from starting the steroid tapering, his fever and abdominal pain recurred afterward. Again, blood tests were abnormal as follows: elevated levels of WBCs (15000 /μL with 94.1 % neutrophils), CRP (12.7 mg/dL), HbA_1c_ (9.5 %), but normalized IgG4 (84.5 mg/dL). CT revealed an extensive low-density area in the hepatic segment 7–8 (Fig. 1b). The lesion was encapsulated by an enhanced wall and contained gas and fluid. It was diagnosed as a hepatic abscess. As we considered the liver abscess caused by the steroid therapy, we continued to taper steroid amount and stopped in one month after diagnosis of the hepatic abscess. US-guided percutaneous abscess drainage was performed, as well as antibiotic treatment with ampicillin-sulbactam was started with 13.5 g of daily dose and continued for 10 days. *Klebsiella pneumoniae* was isolated from both liver aspirates and blood culture. Although his laboratory data improved and the patient was discharged in 10 days, complete liquefaction of the IPT component needed time and the abscess was refractory. Percutaneous drainage was finally withdrawn after confirming the shrinkage of the abscess at the sixth month (Fig. 1c), and no sign of recurrence was seen for the following 16 months.

## Conclusion

In general, hepatic IPTs are difficult to differentiate from malignant tumors, as their incidence is rare, their pathogenesis is diverse, and imaging findings are varied [1–4, 6, 7, 11]. In some cases, the images of hepatic IPTs resemble metastases from distant origin tumors, such as pancreatic cancer [12] and malignant gastrointestinal stromal tumors [13]. In contrast, some of the hepatic tumors mimicking IPT in cases with IgG4-RD have been revealed to be a recurrence of long-time latent cancer confirmed by a percutaneous biopsy [14]. Hence, the histological confirmation of the hepatic mass is critical, especially in cases with IgG4-RD or those suspected of being hepatic IPT. One concern in percutaneous liver biopsy is needle-tract seeding in patients with malignant tumors, which is recognized in 2.7 % of cases [15]. Therefore, caution is needed for the selection of biopsy targets and unnecessary biopsies should not be performed, for instance in cases of doubtless cancers presumed to be curative by resection.

From the viewpoints of differentiating their pathogenesis or subtypes, biopsy is recommended in cases of a suspected hepatic IPT. As described above, this tumor is caused by a variety of pathogeneses [1–7, 11], and their histology is quite distinctive. For instance, follicular dendritic cell tumor is specific to Epstein-Barr virus infection [1]; tuberculosis [3] is detectable by Ziehl-Neelsen staining and typically causes caseating granulomas; and inflammatory myofibroblastic tumor is typically seen in the lung and liver [16]. Culture samples can also be obtained by biopsy in cases with a suspected infectious background [2, 3]. In this sense, IgG4-related hepatic IPT features fairly unique histology; that is to say abundant IgG4-positive plasma cell infiltration (>10 cells/HPF in biopsy and >50 cells in surgical materials) [8], storiform fibrosis, and obliterative phlebitis [8, 10, 17]. The sample obtained from current patient contained diagnostic items fulfilling the criteria of possible IgG4-RD [8, 10] and percutaneous needle biopsy is thought to be an effective diagnostic tool for IgG4-related hepatic IPT.

According to a keyword search in PubMed, a total of 11 cases of IgG4-related hepatic IPT were identified [11, 18–23] (Table 1). Including the case in this study, the patients were all men with an average age of 64.8 years (range: 52–77 years). The ratio of a solitary tumor was 91.7 % (11out of 12 cases) and the mean tumor diameter was 3.3 cm (range: 1.4–7.7 cm). The location of the tumors did not show any significant trend. In the cases with available data, the median of serum IgG4 level was 213 mg/dL and the patients showing elevated serum IgG4 (>135 mg/dL) accounted for 85.7 % (6/7). This high incidence of serum IgG4 elevation can be a hint for further examinations of IgG4-RD. In addition, hepatic IPT is not infrequently associated with IgG4-related sclerosing cholangitis, the patients showing sclerosing cholangitis accounted for 50 % (3/6) in our research. Hepatic IPT may represent local inflammatory changes of cholangitis [6]. As most of these cases had already been reported before the publication of a consensus statement on IgG4-RD [8], it is unclear how many of them satisfied the diagnostic criteria [18]. Future study is warranted to clarify the clinical characteristics of IgG4-related hepatic IPT to facilitate its diagnosis.

**Table 1:** Summary of the reported cases of IgG4-related hepaticinflammatory pseudotumor

Author	Year	Age(y.o.)/Sex	Serum IgG4	Number of IPT	Size	Location	Biopsy	Resection	IgG4-RD
Uchida K [19]	2007	54/M	213 mg/dL	1	3 cm	Segment 4	Done		Pancreatitis
Naito I [11]	2009	77/M	231 mg/dL	1	4 cm	Segment 3		done	Sclerosing cholangitis
Kim F [20]	2011	58/M	1470 mg/dL	1	3 cm	Segment 4	Done		Tubulointerstitial nephritis
Horiguchi S [21]	2012	76/M	819 mg/dL	1	1.5 cm	Segment 2	Done		Sclerosing cholangitis
Ahn KS [18]	2012	58/M	NA	1	3 cm	NA		done	NA
Ahn KS [18]	2012	60/M	NA	1	7.7 cm	NA		done	NA
Ahn KS [18]	2012	76/M	NA	2	2.3 cm	NA	Done		NA
Ahn KS [18]	2012	52/M	NA	1	4 cm	NA	Done		NA
Lee YS [22]	2013	59/M	75 mg/dL	1	NA	Segment 5	Done		Sclerosing cholangitis
Matsuo Y [13]	2014	74/M	NA	1	1.4 cm	Segment 8		done	NA
Yang L [23]	2015	60/M	159 mg/dL	1	NA	Segment 3	Done		Esophageal mass, gastric ulcer
Our Case	2015	73/M	137 mg/dL	1	3 cm	Segment 7–8	Done		None

*M* male, *NA* data not available

To date, adverse events related to corticosteroid therapy for IgG4-RD have rarely been reported. In the present case, several concerns were raised about steroid treatment, such as worsening blood glucose control, immunocompromisation, obesity, osteoporosis, ophthalmologic diseases (cataract and glaucoma) and steroid withdrawal syndrome. In autoimmune pancreatitis or one of the most popular subtypes of IgG4-RD, many cases actually show improvement in diabetes by steroid treatment [24], as they are cases of pancreatic diabetes. The current patient with diabetes and immunocompromised condition is at an increased risk of developing a pyogenic hepatic abscess [25]. This abscess, which originally consisted of dense fibrous tissue, took a long time (six months) to resolve, probably due to associated diabetes, immunocompromised status, and slow liquefaction of the mass. The clinician must be cautious about the possible adverse events associated with corticosteroids for the treatment of IgG4-related hepatic IPT.

The diagnosis of hepatic IPT is difficult, especially in terms of differentiation from malignant tumors. Histological diagnosis is essential and percutaneous needle biopsy is effective for the diagnosis of IgG4-related IPT. Clinicians must bear in mind that corticosteroid treatment is not always ideal and may cause critical adverse events in cases with IgG4-RD.

## Abbreviations

CT, computed tomography; HbA1c, hemoglobin A1c; HPF, high power field; IgG4, immunoglobulin G4; IgG4-RD, IgG4-related disease; IPT, inflammatory pseudotumor; US, ultrasonography
